# Integration of Human Walking Gyroscopic Data Using Empirical Mode Decomposition

**DOI:** 10.3390/s140100370

**Published:** 2013-12-27

**Authors:** Vincent Bonnet, Sofiane Ramdani, Christine Azevedo-Coste, Philippe Fraisse, Claudia Mazzà, Aurelio Cappozzo

**Affiliations:** 1 Movement to Health (M2H) Laboratory, EuroMov, University of Montpellier 1, Montpellier 34090, France; E-Mail: sramdani@univ-montp1.fr; 2 LIRMM, University of Montpellier 2, Montpellier 34090, France; E-Mail: fraisse@lirmm.fr; 3 INRIA, DEMAR-LIRMM, Montpellier 34090, France; E-Mail: azevedo@lirmm.fr; 4 Department of Mechanical Engineering, University of Sheffield, Sheffield S13JD, UK; E-Mail: c.mazza@sheffield.ac.uk; 5 Department of Movement, Human and Health Sciences, University of Rome “Foro Italico”, Rome 00135, Italy; E-Mail: aurelio.cappozzo@uniroma4.it

**Keywords:** empirical mode decomposition (EMD), inertial measurement unit (IMU), human walking, motion analysis

## Abstract

The present study was aimed at evaluating the Empirical Mode Decomposition (EMD) method to estimate the 3D orientation of the lower trunk during walking using the angular velocity signals generated by a wearable inertial measurement unit (IMU) and notably flawed by drift. The IMU was mounted on the lower trunk (L4-L5) with its active axes aligned with the relevant anatomical axes. The proposed method performs an offline analysis, but has the advantage of not requiring any parameter tuning. The method was validated in two groups of 15 subjects, one during overground walking, with 180° turns, and the other during treadmill walking, both for steady-state and transient speeds, using stereophotogrammetric data. Comparative analysis of the results showed that the IMU/EMD method is able to successfully detrend the integrated angular velocities and estimate lateral bending, flexion-extension as well as axial rotations of the lower trunk during walking with RMS errors of 1 deg for straight walking and lower than 2.5 deg for walking with turns.

## 1. Introduction

The last decade has seen the development of inertial measurement units (IMUs) to measure human movement performance [1]. Mass-market electronics companies provide low cost inertial devices, such as those contained in cellphones, that are easy to use, sturdy and small. One of the main advantages is that monitoring movement outside of a laboratory and for a prolonged period of time is now possible [2]. However, with the decrease in size and price, the accuracy of these sensors has correspondingly declined, leading to the main drawback of low cost IMUs, *i.e.*, sensor drift. Sensor drift is a non-linear and low frequency time-varying offset that is due to manufacturing inaccuracies, temperature changes or ageing of electronic components [3]. An IMU usually measures three accelerations and three angular velocities which, in principle, enables operators to estimate the position and orientation through double and single integration of these signals, respectively. Unfortunately, drift jeopardizes the time integration of raw signals [4]. The resulting integrated signals, especially for prolonged recordings, can then be flawed with a very substantial non-linear trend, thus hampering their use.

To overcome these issues, Kalman recursive filters are often used in the literature for real-time estimates of the IMU orientation by fusing accelerometer and gyroscope data. An accurate estimate of only two orientation angles (pitch and roll angles) can be obtained with this approach [3,5], unless magnetometers are used as an additional source of information. Recently, the use of a Weighted Fourier Linear Combiner (WFLC) adaptive filter proved to be very accurate in estimating real-time 3D lower trunk angles from angular velocity data during treadmill walking [6]. However, being based on Fourier series decomposition, the WFLC approach is limited to quasi-periodic signals. In addition, the WFLC algorithm requires *a priori* knowledge of the shape and characteristics of the measured signal and its associated noise. It should also be noted that the effectiveness of these methods depends on the correct choice of their internal parameters, which might vary for different motor tasks or populations [5,6].

As stated previously, a marked non-linear trend is observed in integrated IMU raw data [4]. Huang *et al.* [7] introduced the empirical mode decomposition (EMD) approach, which has received increasing attention for trend estimation in the signal processing community [8,9]. The EMD method has been successfully applied to estimate trends in long-term non-stationary and non-linear signals in numerous engineering and biomedical fields [8]. As for Fourier decomposition or wavelet transforms, EMD decomposes a signal into a set of basis functions. This method is fully data-driven and local in time, thus allowing identification of non-linear trends at different time scales. The EMD method only requires an oscillatory pattern, not signal periodicity. When dealing with integrated signals, the unwanted non-linear trend due to sensor drift is identified as the low frequency component, while the oscillatory subject motion is the high frequency component. Recently, extended versions of the EMD method have been used to denoise angular velocity generated by gyroscope sensors, although not during human motion [10,11]. These methods, however, require the tuning of several internal parameters and did not focus on angular velocity integration. Due to its empirical nature, the EMD algorithm does not admit an analytical formulation that could allow theoretical analysis [9]. Consequently, this paper investigated experimentally the EMD capabilities in detrending integrated data obtained from one IMU, placed at the lower trunk level, and thereafter estimating its 3D orientation, during overground and treadmill walking at steady-state and transient speeds and direction of progression of abled-bodied volunteers. The 3D lower trunk orientation was chosen as a paradigmatic case because it appears to be a crucial variable to monitor and evaluate motor capacity and performance of individuals with different age or health status [12–16].

## 2. Methods

### 2.1. Empirical Mode Decomposition

**Figure 1.**
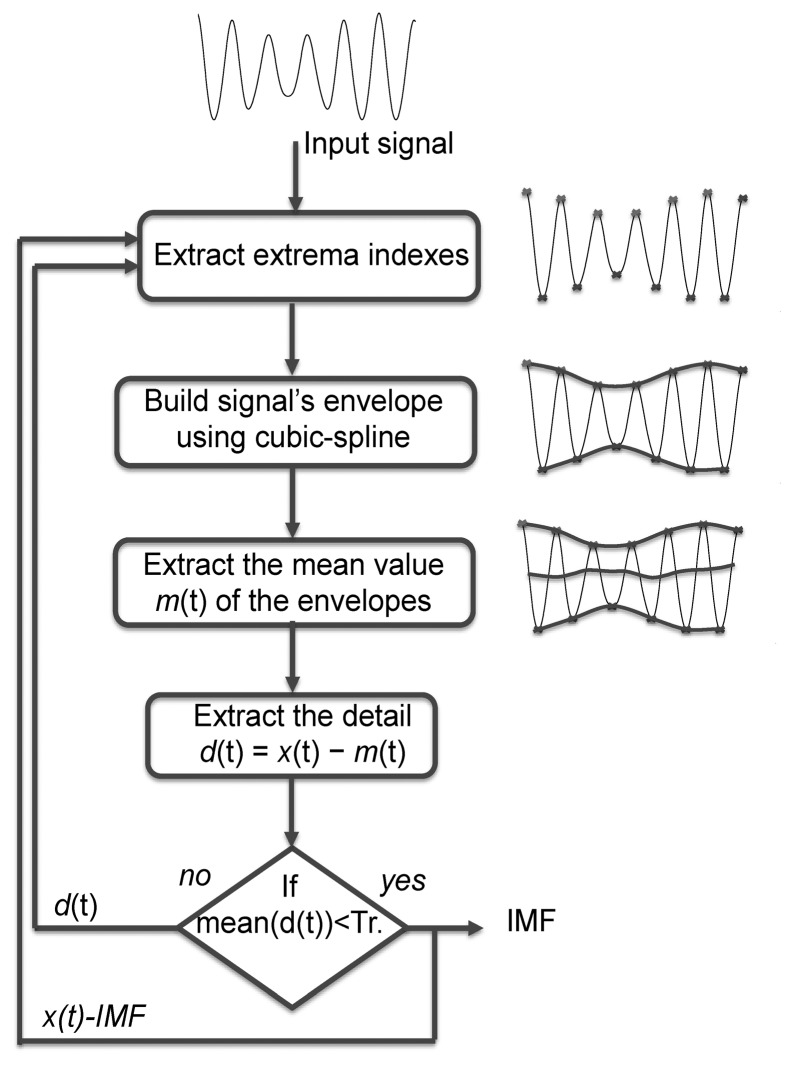
Block diagram summarizing the basic EMD steps.

Empirical mode decomposition (EMD) is an adaptive method for data analysis [7]. EMD is based on the assumption that the analysed signal is composed of superimposed fast and slow oscillations. In this context, the signal is considered at the local oscillation scale. Note that EMD does not require selection of any input parameters or initial conditions. Interested readers will find further details on EMD and its different implementations in [7–9]. The first step of the EMD algorithm is to detect extrema in a given signal *x*(*t*). In the second step, using cubic-spline interpolation, two envelopes are built from these minima and maxima. In the third step, the mean value *m*(*t*) of the envelop is calculated. The fourth step involves subtracting this mean value from the original signal in order to obtain the detail *d*(*t*) = *x*(*t*) − *m*(*t*). These four steps are iterated through the so-called sifting process [7] until the signal *d*(*t*) can be considered as zero-mean, *i.e.*, the mean value of *d*(*t*) is below a user-specified threshold (*Tr*), which defines a stopping criterion. The threshold selection issue has already been addressed [7,8] and here we used the typical value (0.05) recommended in [8]. The resulting detail is called an Intrinsic Mode Function (IMF). The previous steps, including the sifting process, are then iterated on the detailed signal *d*(*t*). The EMD basic steps are shown in Figure 1. The final decomposition of *x*(*t*) reads:

(1) $$\text{X}(\text{t})=\sum_{\text{k}=1}^{\text{D}}{\text{d}}_{\text{k}}(\text{t})+\text{r}(\text{t})$$

where *d_k_*(*t*) are the *D* successive IMFs and *r*(*t*) is the final residual. IMFs are oscillatory signals that are locally zero-mean [7]. The residual *r*(*t*) is the low-frequency mean trend. Note that all IMFs, except *r*(*t*), are mean stationary. The higher order IMF index, *i.e.*, *D*, is found by the algorithm itself and is signal-dependent. In this paper, the standard implementation proposed by Flandrin *et al*. [8] was used [17].

### 2.2. Detrending and Orientation Estimate

The gyroscopes embedded in the IMU measure the angular velocity components that allow estimation of the change in orientation by integration [18]. Each of the three angular velocities of the IMU sensor were thus integrated separately using Simpson's numerical approximation along their corresponding sensor axes. These integrated signals do not necessarily coincide with the actual rotations of the corresponding axis of the IMU local frame. This is because a small rotation of the IMU local reference system occurs at each instant relative to the previous instant [6,18]. At each time point these rotations were estimated by using the integrated signals to calculate a rigid transformation matrix [18]. The resulting quantities, that have the dimensions of angles, served as signal inputs for the EMD algorithm that estimates trends due to the integration error and sensor drift. Each of the estimated trends was then subtracted from the corresponding angle time histories in order to obtain the orientation angles.

As specified by Flandrin *et al*. [8], the trend is expected to be captured by IMFs of large indices in addition to the final residual. In fact, due to the algorithm formulation, the IMF frequency content decreases while the IMF index increases contrary to the IMF amplitude. Note that, due to the empirical nature of the method, the number of IMFs is sensitive to the length of the analysed samples [19] and to the signal shape. In order to provide a general criterion that helps determining whether an IMF should be used in the trend estimate or not, Flandrin *et al*. [8] and Rilling *et al*. [9] proposed the following criterion, which is called the mean cumulative sum (*MCS*):

(2) $$\text{MCS}(\text{D})=\frac{1}{\text{N}}\sum_{\text{t}=1}^{\text{N}}(\sum_{\text{k}=1}^{\text{D}}{\text{d}}_{\text{k}}(\text{t}))$$

where *N* is the total number of recorded samples during a given trial. Since the lowest IMFs correspond to the high frequency and small amplitude signal components, their contribution to *MCS* is expected to be relatively small. The contribution of the largest IMFs, relating to low frequency and large amplitude components, will induce a large and abrupt gap into the *MCS* value. Flandrin *et al*. [8] suggested that the last IMF before the abrupt gap, annotated as IMF_b_, should be selected in addition to the largest IMF for the trend estimate (see Figure 4).

To assess the relevance of this approach for applications in human walking analysis, we computed the root mean square (RMS) difference between the angles estimated through the EMD method and the angles provided by a stereophotogrammetric system, used as reference system, for different IMF indexes. The RMS difference was first calculated using only the final residual as trend, then using all IMFs with an index greater than IMF_b_, and finally adding the IMF occurring just before IMF_b_.

### 2.3. Data Collection

A total of 30 volunteers (16 males, 14 females, age range: 22–60 years, stature: 1.75 ± 0.08 m, mass: 75 ± 10 kg) were included in the study after signing an informed consent. They were randomly separated into two different groups that executed a number of different walking tasks.

The goal of these experiments was to collect IMU signals during walking for as many walking situations as possible. Both treadmill and overground gait were investigated for different walking speeds, transient and 180 deg turning phases and steady-state conditions.

The first group of 15 subjects was asked to perform three walking trials at: (1) natural walking speed; (2) 80% and (3) 120% of natural speed on a motorized treadmill for 80 s. The subjects initially stood on the stationary treadmill, which was then accelerated to the desired velocity for 35 s. Thereafter, the treadmill was decelerated and stopped for 5 s before being reaccelerated up to the same velocity. The stopping phase was used to assess the ability of the proposed algorithm to provide accurate estimates also during non-oscillating motion over a short time interval.

The second group of 15 subjects was asked to walk up and down for three times along a 12-m rectilinear walkway at self-selected speed. These experiments were included to mimic a more ecological type of walking with respect to the one observable on the treadmill [20] and to involve 180 deg turning phases and to experimentally show the robustness of the proposed algorithm.

An IMU (Sensorize SRL, Freesense^©^, Rome, Italy) was located on the lower back (L4-L5) of the subjects so that the unit local frame axes were aligned with the anatomical axes of the lower trunk. In addition, three retro-reflective markers were attached to the IMU sensor in order to define a marker-cluster local frame. During the above-mentioned walking trials, the angular velocities were collected from the IMU (*f_s_* = 100 samples·s^−1^) and the marker trajectories were tracked by eight infrared cameras (MX, Vicon^©^, Oxford, UK, *f_s_* = 100 samples·s^−1^). Pitch, roll and yaw angles, describing the orientation of the IMU frame, were estimated from the IMU data using the EMD algorithm and those describing the orientation of the marker cluster frame were reconstructed using the data provided by the stereophotogrammetric system. The time invariant offset of the marker cluster frame orientation relative to the IMU frame orientation was mathematically removed through a rigid transformation calculated while the subject was standing still. In this way, both instruments were assumed to provide the same pitch, roll, and yaw angles.

### 2.4. Accuracy of the Orientation Estimate

The accuracy of the EMD in the pitch, roll and yaw angle estimation was assessed by comparing these angles to the corresponding angles obtained from the stereophotogrammetric data in the two walking conditions. The RMS and correlation coefficient (*CC*) between the estimated and measured angles were calculated for the whole trial set for the treadmill data. Concerning overground walking, the EMD was applied, first over the entire dataset collected by the IMU and, secondly over a smaller sub-dataset. The entire dataset includes turning phases, steady-state walking and acceleration-deceleration phases, whereas the smaller sub-dataset corresponds to the parts of the recording when the subject was walking steady-state within the stereophotogrammetric capture volume (four steps). Performing the analysis on the entire data set and on smaller epochs allows assessing the robustness of the IMU/EMD method with respect to the turning phases and with respect to the number of considered samples.

## 3. Results

**Figure 2.**
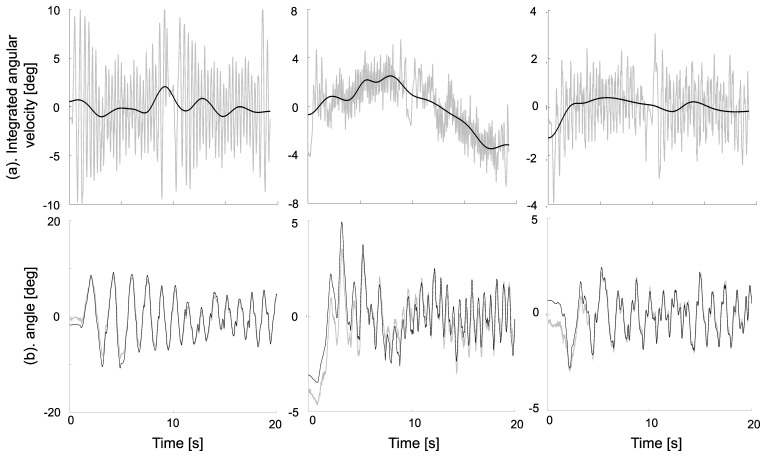
Data relative to the pitch, roll and yaw angles obtained for one randomly selected treadmill walking trial. The integrated angular velocities (grey line) and the resulting trends (black line) are estimated using EMD (**a**) during all the trial; zoom over 20 s on the corresponding detrended angles are thereafter estimated (black line) and compared with those obtained using stereophotogrammetry (grey line) (**b**).

The ability of the EMD method to detrend the integrated angular velocity components and thus to estimate the sensor orientation is depicted in Figure 2, showing the pitch, roll and yaw angles during an entire randomly selected treadmill walking trial.

**Figure 3.**
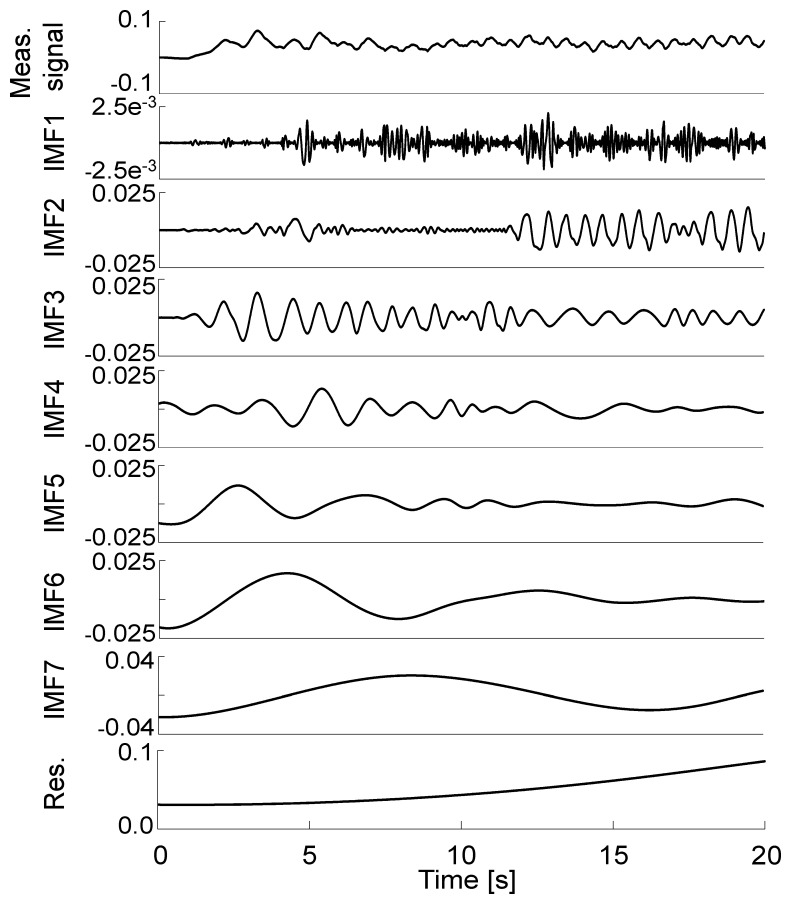
Representative normalized IMF decomposition of the integrated angular velocity obtained for the first 20 s of one randomly selected treadmill walking trial. All the IMFs are expressed in rad.

Figure 3 represents the signal decomposition into IMF of the corresponding roll angle. This figure illustrates the quantitative difference between the level of high frequency components, which are contained in the first IMFs, and the components identified as drift, *i.e.*, IMF 7 and the final residue.

**Figure 4.**
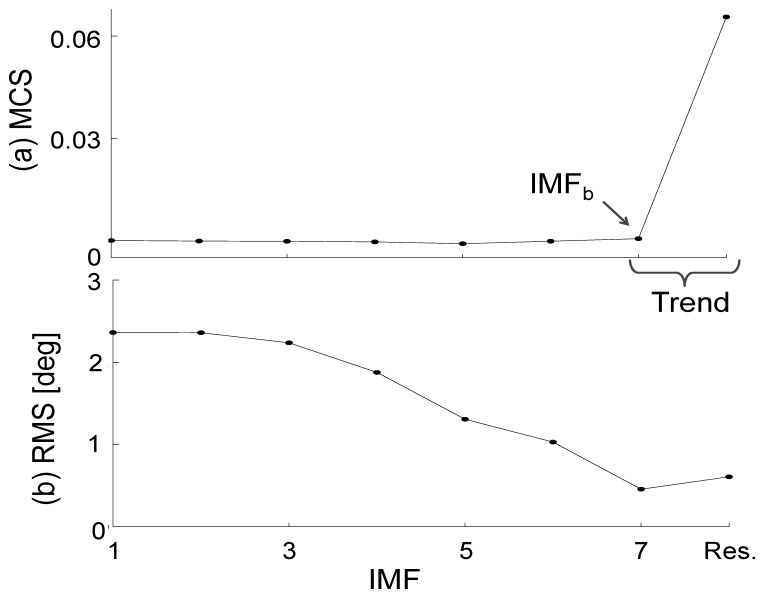
Representative results obtained for one randomly selected trial showing the variations in the retained criterion (Equation (2)) used to select the number of IMFs used in the trend estimate (**a**). In this trial, IMF number seven was selected as the best one for trend estimation (**b**).

The *MCS* values used to select the IMF in the trend estimate are shown in Figure 4a together with the corresponding RMS difference for a randomly selected trial (Figure 4b). As expected, IMF_b_ was the mode that allowed the best detrending of the integrated angular velocity.

**Figure 5.**
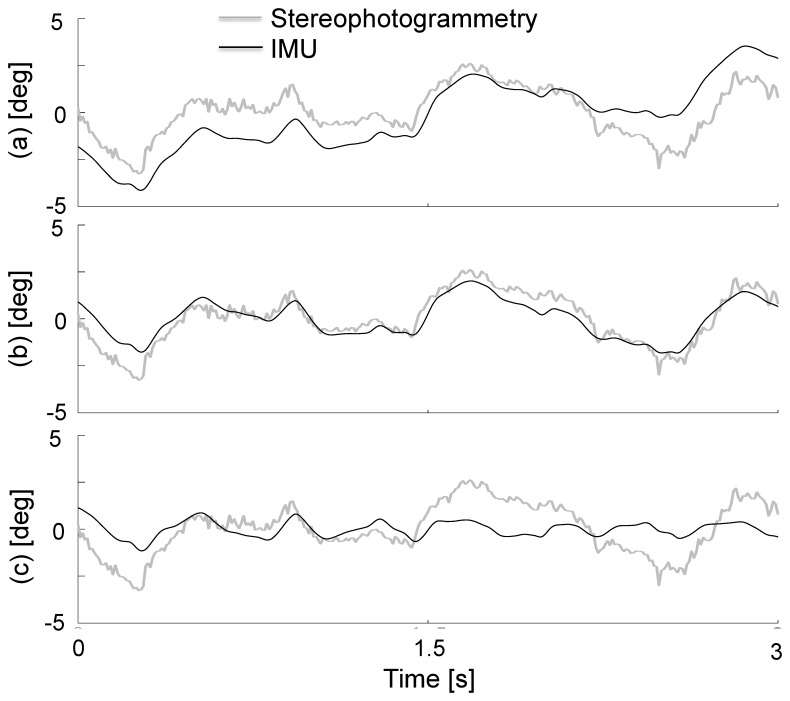
Roll angle, during a randomly selected overground steady-state walking trial, using a trend estimate of the integrated angular velocity using only the final residual (**a**), when IMF_b_ is also removed (**b**) and when too many IMFs are removed (**c**).

Figure 5 shows a comparison between the roll angle time histories during overground walking as obtained using IMU data and the EMD method with different IMF indexes, and stereophotogrammetric data. These results clearly show that when too many IMFs are removed the trend would tend to reduce the amplitude of the estimated angle.

The results of the proposed method are presented in Tables 1 and 2 for the treadmill and overground walking trials, respectively. The outputs of the proposed method are presented in the fourth column (IMF_b_) of these tables, which correspond to the correct identification of the number of IMFs used in detrending the integrated angular velocities. The average RMS was less than 1 deg for treadmill walking and less than 2.1 deg for overground walking for the yaw, pitch and roll angles. The average correlation coefficient was above 0.7 for all of the investigated angles.

Moreover, the two tables present the results of a sensitivity analysis concerning the choice of the number of IMFs required for the accurate detrending of integrated angular velocities. As expected and presented in these tables, the best results were obtained when the final residual and the consecutive IMF up to IMF_b_ were used to detrend the integrated angular velocities (IMF_b_ column). The selection of the relevant number of IMFs is crucial, as already illustrated by Figure 5. As illustrated by the Final residual column, if the trend is underestimated for overground walking, by selecting only the final residual, very large RMS differences can be obtained. These large RMS differences, *i.e.*, up to 6.2 deg and 2.9 deg for yaw and roll angles, highlight the influence of a not sufficiently compensated cumulative drift. Indeed after 80 s of overground walking, the cumulative error due to incorrect compensation of the drift can be very substantial. Note that without the use of any compensation, with the data collected for overground walking, for the yaw angle, the RMS difference may be up to 18 deg.

## 4. Conclusions

The aim of this study was to validate a method based on Empirical Mode Decomposition, to obtain a drift-free estimate of the 3D orientation of a gyroscope sensor attached to the lower trunk during walking.

A sensitivity analysis was performed to determine the number of Intrinsic Mode Functions that should be used to detrend integrated angular velocities. The results of this analysis, as presented in Tables 1 and 2, showed that the criterion (Equation (2)) proposed by Flandrin *et al*. [8] can be used to effectively predict the number of relevant IMFs required for the detrending. By selecting the last IMF, called IMF_b_, before the break in the *MCS* value (see Figure 4) the method proved to be very accurate in estimating all three angles, as represented in Figure 2, and this was the case not only during steady-state walking but also when turning and acceleration-deceleration phases occurred. The ability of the method to generate accurate angle estimations during oscillating but non-periodic motions paves the way to future applications for motor tasks other than walking.

The accuracy of the estimates of lower trunk bending in the sagittal (pitch) and frontal (roll) planes is similar to that obtained using a properly optimized Kalman filter [5] and the accuracy of the estimate of trunk rotation (yaw) is similar to that obtained using a model based on a WFLC recursive filter [6]. In addition, an advantage of the adaptive filter approaches is that, contrary to the proposed method, they are suitable for real-time applications [3,5,6]. However, the Kalman filter, despite fusing 3D gyroscopic and accelerometric data, is unable to estimate the yaw angle. Conversely, it is very robust regarding the signal shape with an RMS error of 0.6 and 0.5 deg for pitch and roll angles respectively [5]. This algorithm requires basic knowledge on the sensor noise level to fuel the covariance matrix used in the prediction-estimation process [3]. Moreover, fine weighting of five internal gains of the filter is required to obtain accurate estimates [5]. The WFLC algorithm, that only uses gyroscopic data as input, has been assessed for overground steady-state walking in both able-bodied and pathological subjects [6,15]. It allowed an estimate of yaw, pitch and roll with RMS errors of 1.1, 0.8 and 0.4 deg. However, this method is highly dependent on the signal shape and it is meant to be used only for quasi-periodic signals [4,6,21]. In addition, it requires fine-tuning of four gains and of the initial filter weights, *i.e.*, the choice of the first guess of the frequency and amplitude of the measured signal, which depends on the specific task analysed. In summary, the choice of one among the several possible methods depends on the application, the associated constraints, and accuracy needs.

Recently, online versions of the EMD have been proposed [9,22,23]. However, these implementations cannot be qualified as real-time since they introduce a delay due to the use of a sliding window moving over the signal. In addition, they require the tuning of more parameters, such as the number of iterations required to extract the IMF or the window size. Finally, offline analysis provides a slightly more detailed decomposition because it even captures very low frequencies [22], which is the aim of our application. Future work will investigate the use of online EMD in integrating IMU data.

It is also important to note that the proposed method was validated with many subjects in very different conditions: treadmill walking, overground walking, different gait velocities, accelerating/decelerating phases or turning phases. These experimental variations were used to test the robustness of the algorithm in real life application conditions. Conceptually, the proposed approach is not limited to lower trunk angle estimates, but could be applied to any segment of the body where “fast” oscillations are superimposed with low frequency components. Moreover, the robustness of EMD to pathological walking, running and other oscillating tasks such as rowing, cycling or swimming should now be addressed.

## Figures and Tables

**Table 1.** Accuracy results obtained during treadmill walking. Final method outputs are in the IMF_b_ column.

**Angles**	**Accuracy**	**Final Residual (***r***)**	**IMF**_b_	**IMF**_b_^−^_1_
Yaw	*RMS* (deg)	1.0 ± 0.3	0.8 ± 0.3	1.2 ± 0.3
	CC	0.94 ± 0.03	0.96 ± 0.02	0.92 ± 0.04
Pitch	*RMS* (deg)	1.1 ± 0.5	1.0 ± 0.4	1.3 ± 0.5
	CC	0.76 ± 0.11	0.82 ± 0.09	0.68 ± 0.12
Roll	*RMS* (deg)	0.5 ± 0.1	0.4 ± 0.1	0.5 ± 0.17
	CC	0.94 ± 0.02	0.95 ± 0.02	0.92 ± 0.03

**Table 2.** Accuracy results obtained during overground walking. Final method outputs are in the IMF_b_ column.

**Angles**	**Accuracy**	**Final Residual (***r***)**	**IMF**_b_	**IMF**_b_^−^_1_
Yaw	*RMS* (deg)	6.2 ± 5.5	2.1 ± 1.3	2.9 ± 1.1
	CC	0.41 ± 0.32	0.68 ± 0.23	0.44 ± 0.27
Pitch	*RMS* (deg)	1.0 ± 0.5	1.0 ± 0.4	1.0 ± 0.3
	CC	0.76 ± 0.11	0.79 ± 0.34	0.64 ± 0.27
Roll	*RMS* (deg)	2.9 ± 1.1	1.0 ± 0.5	1.9 ± 0.8
	CC	0.76 ± 0.18	0.71 ± 0.22	0.34 ± 0.27

## References

[1] Fong D.T.P., Chan Y.Y. (2010). The use of wearable inertial motion sensors in human lower limb biomechanics studies: A systematic review. Sensors.

[2] Chelius G., Braillon C., Pasquier M., Horvais N., Pissard Gibollet R., Espiau B., Azevedo-Coste C. (2011). A Wearable sensor network for gait analysis: A six-day experiment of running through the desert. IEEE/ASME Trans. Mechatron..

[3] Haid M., Breitenbach J. (2004). Low cost inertial orientation tracking with Kalman filter. Appl. Math. Comput..

[4] Tan U.X., Veluvolu K.C., Latt W.T., Shee C.Y., Riviere C.N., Ang W.T. (2008). Estimating displacement of periodic motion with inertial sensors. IEEE Sens. J..

[5] Mazzà C., Donati M., McCamley J., Picerno P., Cappozzo A. (2011). An optimized Kalman filter for the estimate of trunk orientation from inertial sensors data during treadmill walking. Gait Posture.

[6] Bonnet V., Mazzà C., McCamley J., Cappozzo A. (2013). Use of weighted Fourier linear combiner filters to estimate lower trunk 3D orientation from gyroscope sensors data. J. NeuroEng. Rehabil..

[7] Huang N.E., Shen Z., Long S.R., Wu M.L., Shih H.H., Zheng Q., Yen N.C., Tung C.C., Liu H.H. (1998). The empirical mode decomposition and Hilbert spectrum for nonlinear and non-stationary time series analysis. Proc. R. Soc. Lond. A.

[8] Flandrin P., Goncalvès P., Rilling G. Detrending and Denoising with Empirical Mode Decomposition.

[9] Rilling G., Flandrin P., Goncalvès P. On Empirical Mode Decomposition and Its Algorithms.

[10] Qian L., Xu G., Tian W., Wang J. (2009). A novel hybrid EMD-based drift denoising method for a dynamically tuned gyroscope (DTG). Measurement.

[11] Zhang Y., Wang S., Xia D. EMD-Based Denoising Methods in the MEMS Gyroscope De-Drift.

[12] Iosa M., Mazzà C., Pecoraro F., Aprile I., Ricci E., Cappozzo A. (2010). Control of the upper body movements during level walking in patients with facio scapula humeral dystrophy. Gait Posture.

[13] Pecoraro F., Mazzà C., Cappozzo A., Thomas E.E., Macaluso A. (2007). Reliability of the intrinsic and extrinsic patterns of level walking in older women. Gait Posture.

[14] Adkin A.L., Bloem B.R., Allum J.H.J. (2005). Trunk sway measurements during stance and gait tasks in Parkinson's disease. Gait Posture.

[15] Mizuike C., Ohgi S., Morita S. (2009). Analysis of stroke patient walking dynamics using a tri-axial accelerometer. Gait Posture.

[16] Grimpampi E., Bonnet V., Taviani A., Mazzà C. (2013). Estimate of lower trunk angles in pathological gait using gyroscope data. Gait Posture.

[17] Empirical Mode Decomposition. http://perso.ens-lyon.fr/patrick.flandrin/emd.html.

[18] Luinge H. (2002). Inertial Sensing of Human Movement. Ph.D. Thesis.

[19] Rilling G., Flandrin P. On the Influence of Sampling on the Empirical Mode Decomposition.

[20] Moseley A., Lanzarone S., Bosman J., Caplan B. (2004). Ecological validity of walking speed assessment after traumatic brain injury: A pilot study. J. Head Trauma Rehabil..

[21] Riviere N., Rader R.S., Thakor N.V. (1998). Adaptive cancelling of physiological tremor for improved precision in microsurgery. IEEE Trans. Biomed. Eng..

[22] Trnka P., Hofreiter M. The Empirical Mode Decomposition in Real-Time.

[23] Guzmán S.A., Fischer M., Heute U., Schmidt G. Real-Time Empirical Mode Decomposition for EEG Signal Enhancement.

